# Supplementary material for: The global emergence of a novel Streptococcus suis clade associated with human infections
Source: EMBO Mol Med. 2021 Jun 17;13(7):e13810. doi: 10.15252/emmm.202013810 (PMC8261479; doi:10.15252/emmm.202013810)
Supplement: Supplementary file 1 — Appendix [file EMMM-13-e13810-s006.pdf]

The global emergence of a novel *Streptococcus suis* clade associated with human infections

Xingxing Dong[1,2#], Yanjie Chao[3#], Yang Zhou[4,5], Rui Zhou[4], Wei Zhang[6], Vincent A. Fischetti[7], Xiaohong Wang[1], Ye Feng[8]*, Jinquan Li[1,4,7]*

Appendix

Appendix Figures S1-S14

Appendix Table S1. Comparison of mortalities in zebrafish inoculated with twenty-five representative *S. suis* isolates from HAC (Human-associated Clade) and HPC (Healthy-pig Clade).

Appendix Table S2. Genes identified special for lineage III.

Appendix Table S3. Genes identified special for lineage II.

Appendix Table S4. Summary of the genome-wide significant and suggestive accessory genes associated with human-associated isolates identified by the GWAS analysis.

Appendix Table S5. Genes identified special for human-associated clade by Chi-square analysis.

Appendix Table S6. Summary of genes affected by 17 missense variants that correlate with human-associated isolates.

Appendix Table S7. Genes identified special for diseased-pig clade.

Appendix Table S8. PCR primers were designed for human-associated *S. suis*.

Appendix Figures

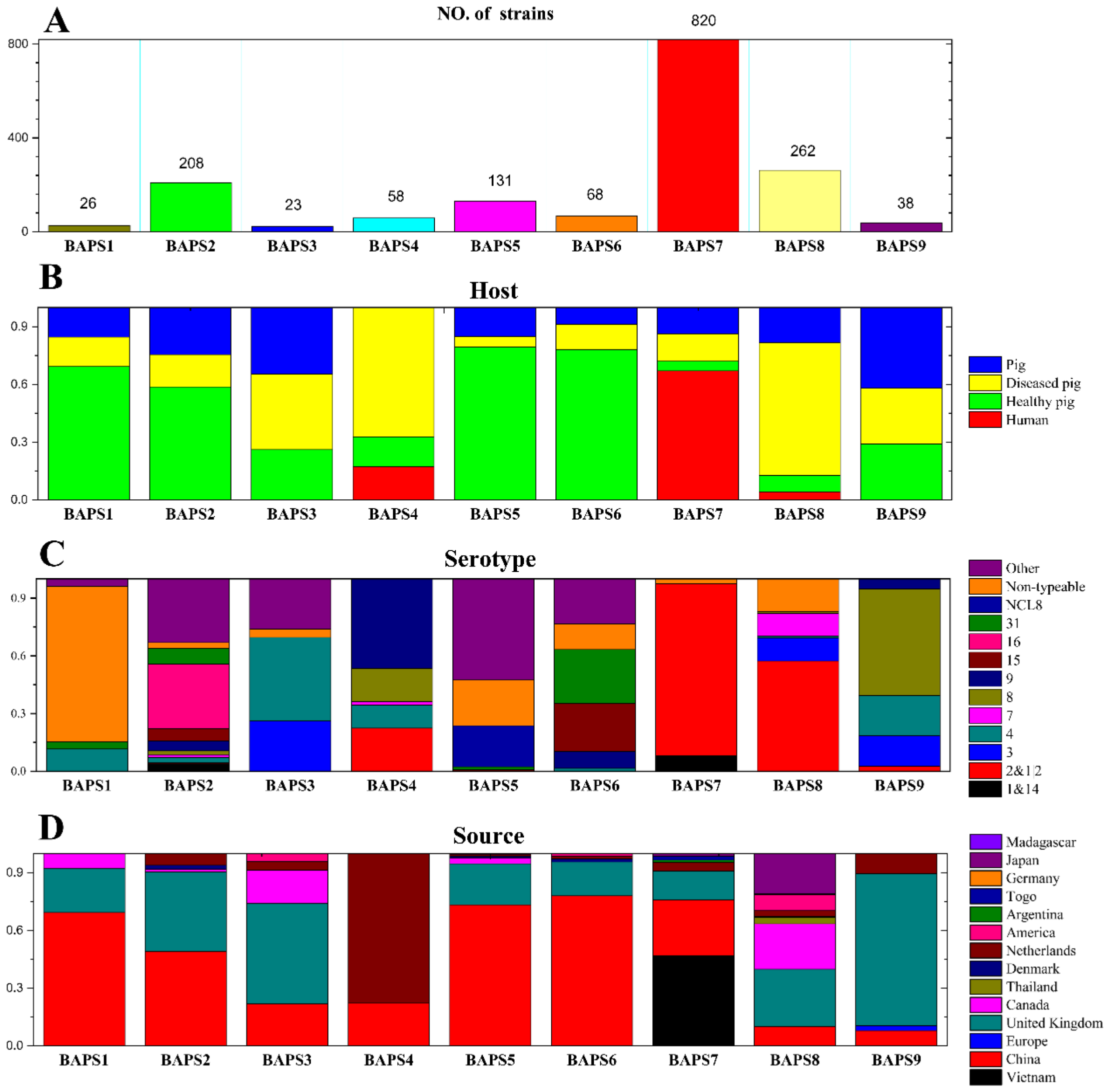

Appendix Figure S1. Characteristics of *S. suis* isolates in BAPS population groups.

A. Number of isolates that were clustered in each of the population groups. B. Fraction of isolates in population groups isolated from different hosts. C. Fraction of serotypes present in each population group. D. Fraction of countries of origin in each population group.

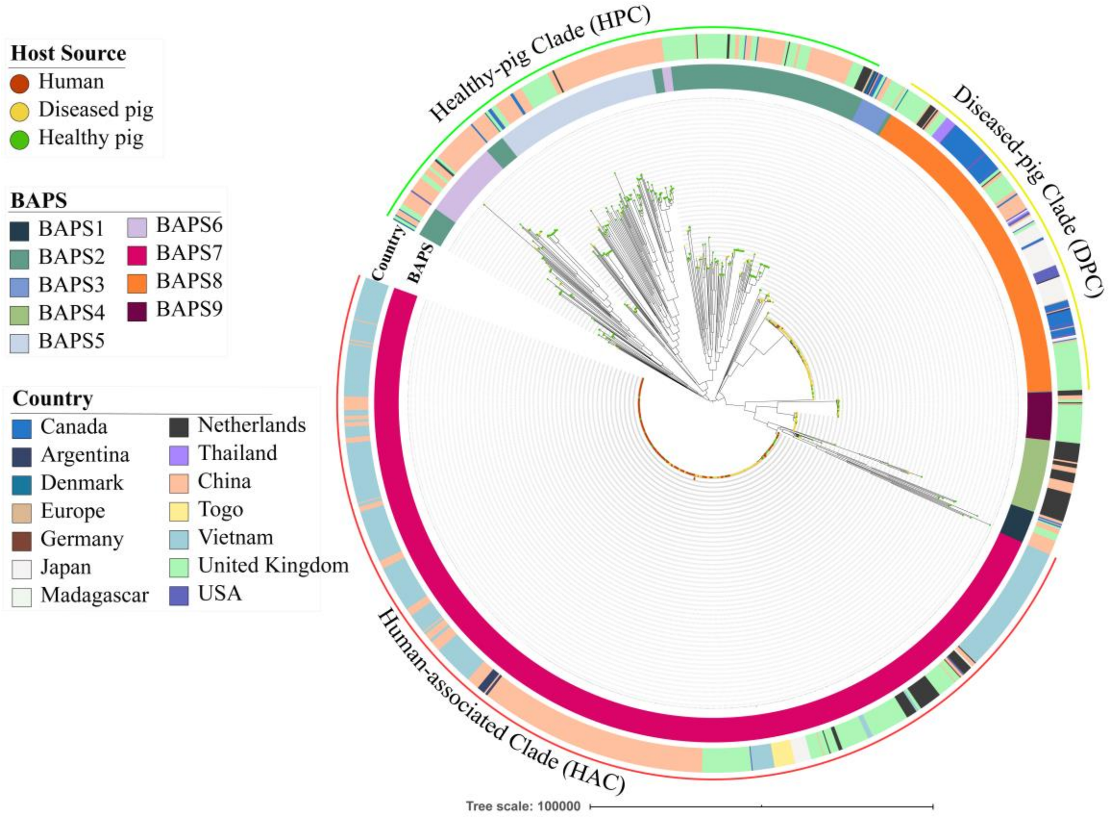

Appendix Figure S2. A midpoint rooted maximum-likelihood tree based on 317,650 non-recombinant genome-wide SNPs from 1634 isolates as determined by Gubbins. Gubbins was run with default parameters. Tip nodes are colored based on the host source (human in red; diseased pig in yellow; healthy pig in green). The rings from inner to outer: the first ring represents BAPS clustering; the second ring represents the origin source. The clades are color coded as follows: Human-associated Clade (HAC) in red, Diseased-pig Clade (DPC) in yellow, and Healthy-pig Clade (HPC) in green.

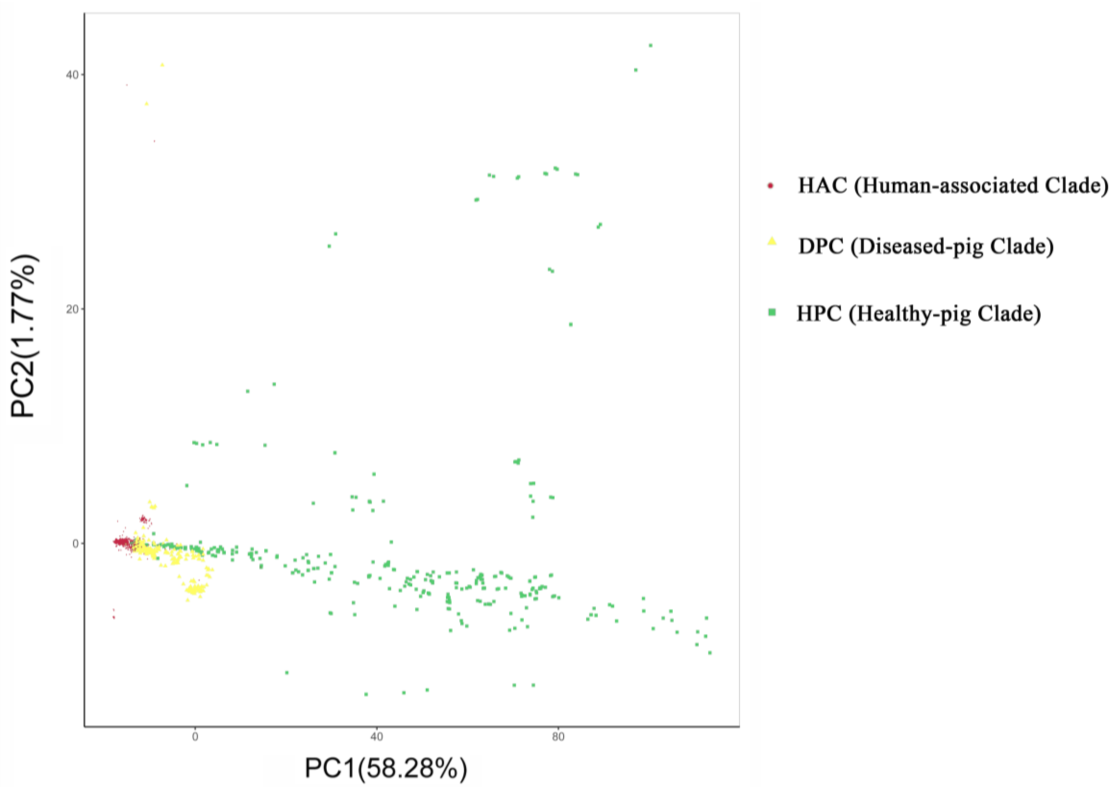

Appendix Figure S3. Principal Components Analysis (PCA) based on the core genome sequence typing. A PCA was performed to examine clustering of 1634 strains using a cgMLST matrix as input. The result was similar to the SNP analyses (Figure 1). Isolates belonging to each clade were identified by a specific color (HAC: red; DPC: yellow; HPC: green). Strains from HPC showed the greatest genetic diversity and some of them tightly clustered with strains in HAC and DPC, respectively.

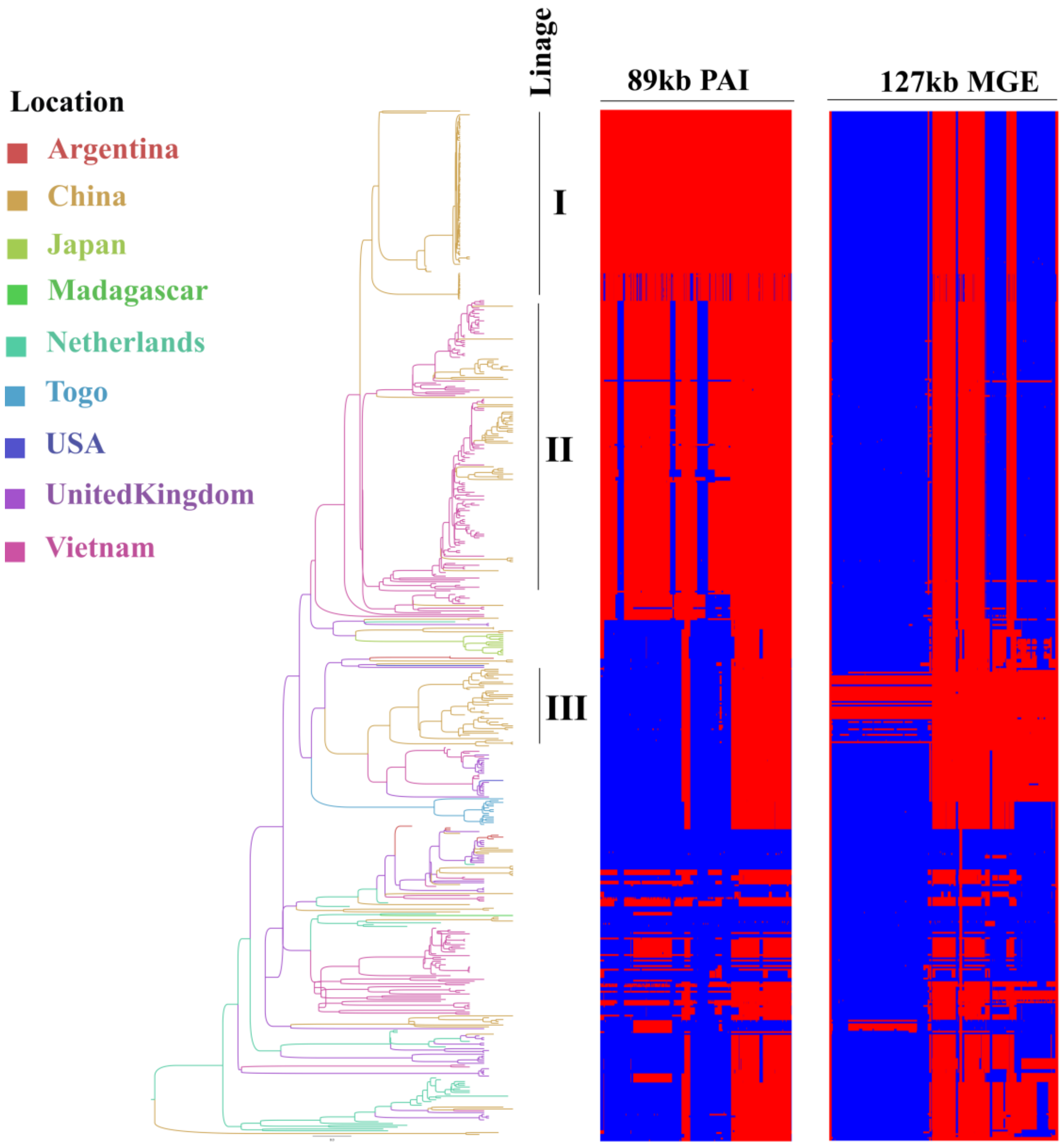

Appendix Figure S4. Phylogeny of 562 *Streptococcus suis* isolates with known collection date from human-associated clade. Maximum clade credibility tree is produced using BEAST2. Major lineages that contain only isolates from Asia are indicated by numbers (I, II, III). *S. suis* genome sequences were mapped against the 89kb pathogenicity island and 127 kb mobile genetic element. The red block of heatmaps represents the presence of 89 kb PAI and 127 kb MGE, the blue represents the absence.

A

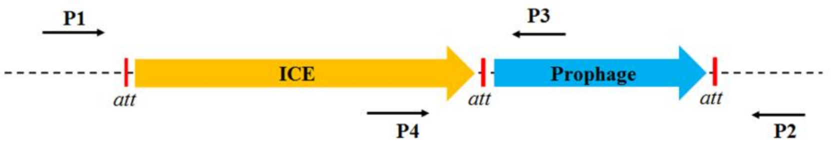

B

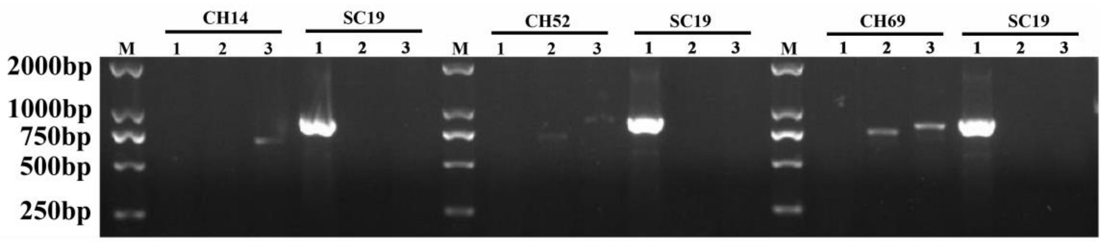

Appendix Figure S5. Confirmation of the site-specific integration and excision of 127-kb mobile genetic element (MGE), integrative conjugative element (ICE) and prophage by PCR. A. The primers used for detection of integrated and excised corresponding element were designed outside of the *att* sites on both ends. The location and orientation of primers are indicated by solid black arrows. Three primer pairs P1/P2, P2/P4 and P1/P3 were used for detection of integrated and excised 127-kb MGE, prophage and ICE, respectively. B. Three specific pairs of primers were applied in each isolate. Each isolate was set to three lanes by the primers in order of P1/P2, P2/P4 and P1/P3. In principle, the PCR-positive result suggests the absence of the corresponding element, whereas the PCR-negative result indicates the presence of the corresponding element. SC19 lacking full length of 127-kb MGE was served as control in each strain. Lane M, 2000-bp ladder. The result confirmed that ICE indeed excise from the chromosome in three isolates, and prophage excise from the chromosome in isolate CH52 and CH69. We did not detect the excision of entire 127-kb MGE.

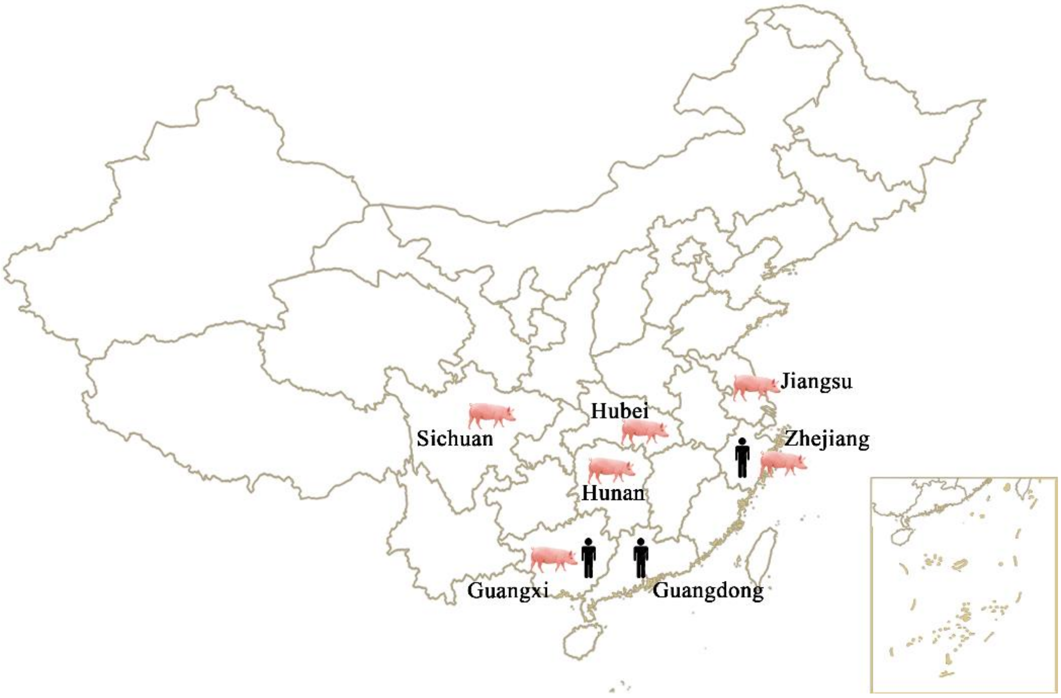

Appendix Figure S6. **Spreading of multi-drug resistant *S. suis* ST7 isolates in China.** Multi-drug resistant *S. suis* ST7 isolates with 127-kb MGE were found in Hunan, Hubei, Guangdong, Guangxi, Sichuan, Jiangsu, Zhejiang province. The host of multi-drug resistant *S. suis* ST7 strains with 127-kb MGE were also shown on the map (pig or human).

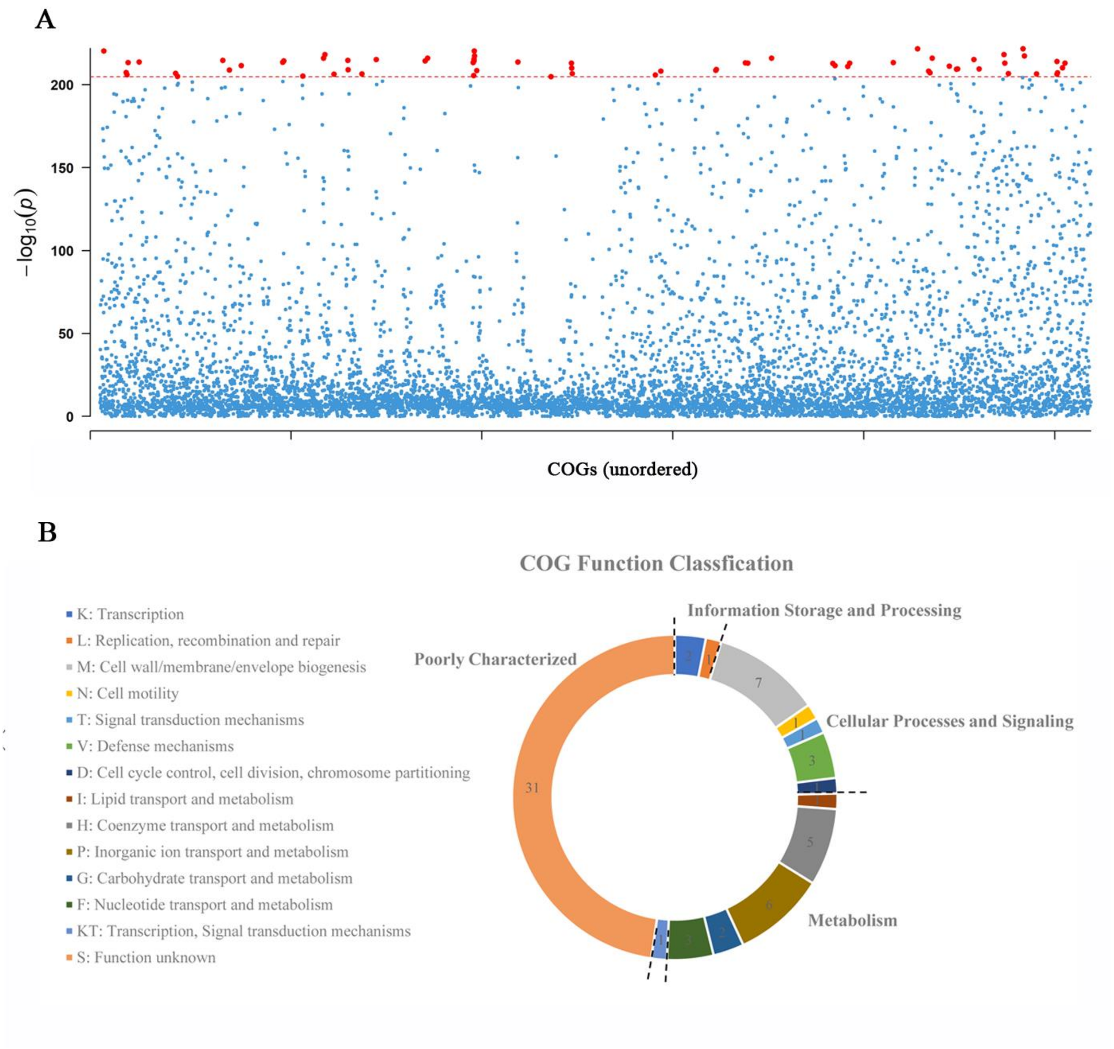

Appendix Figure S7. Accessory genes associated with human-associated isolates. A. show statistical significance (-$\log_{10}$P) for the accessory clusters of orthologous genes (COGs) (unordered) from the pathogen genome-wide association study (GWAS) analysis using GEMMA. The red line designates the genome-wide suggestive P-value thresholds while the accessory genes highlighted in red were identified as significant genes by GEMMA method. B. COG functional classifications of significant accessory clusters of orthologous genes.

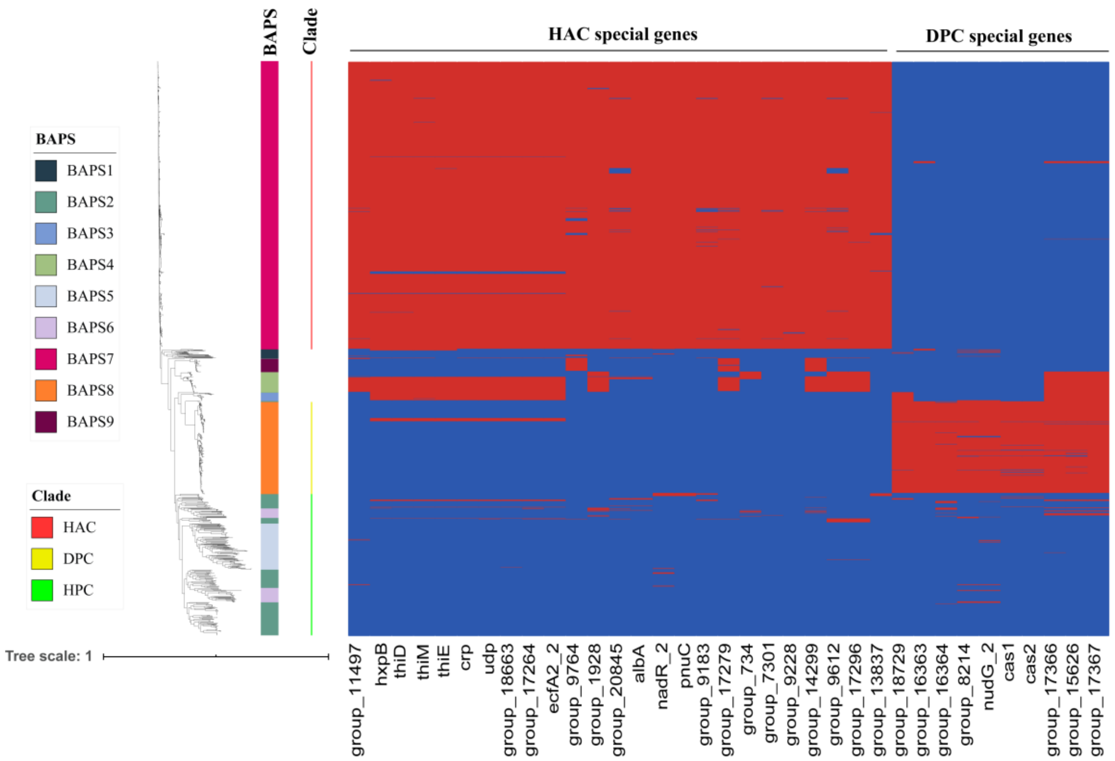

Appendix Figure S8. Pan-genome analysis identified 25 HAC specific genes which might be associated with the evolutionary success of the human-associated clade. Ten genes were also identified to be specific in DPC (Diseased-pig Clade), which provide the evidence that the isolates from diseased pigs in DPC is distinct from the diseased pig isolates in HAC, the latter of which has successfully evolved into human-associated strains. A maximum-likelihood phylogenetic tree was constructed based on whole genome wide SNPs. Clade special genes were screened from accessory genome among the HAC (associated with "Human-associated"), DPC (associated with "Diseased pig"), HPC (associated with "Healthy pig"). Accessory genes leading to a P value $<e^{-30}$ sorted by significance after performing a chi-square test among three clades. Significance genes were further defined as group special genes if their proportion more than 95% in one clade, but less than 5% in other clades. Presence of a gene is indicated in red and absence of a gene in blue.

SC19 (Referenec genome)

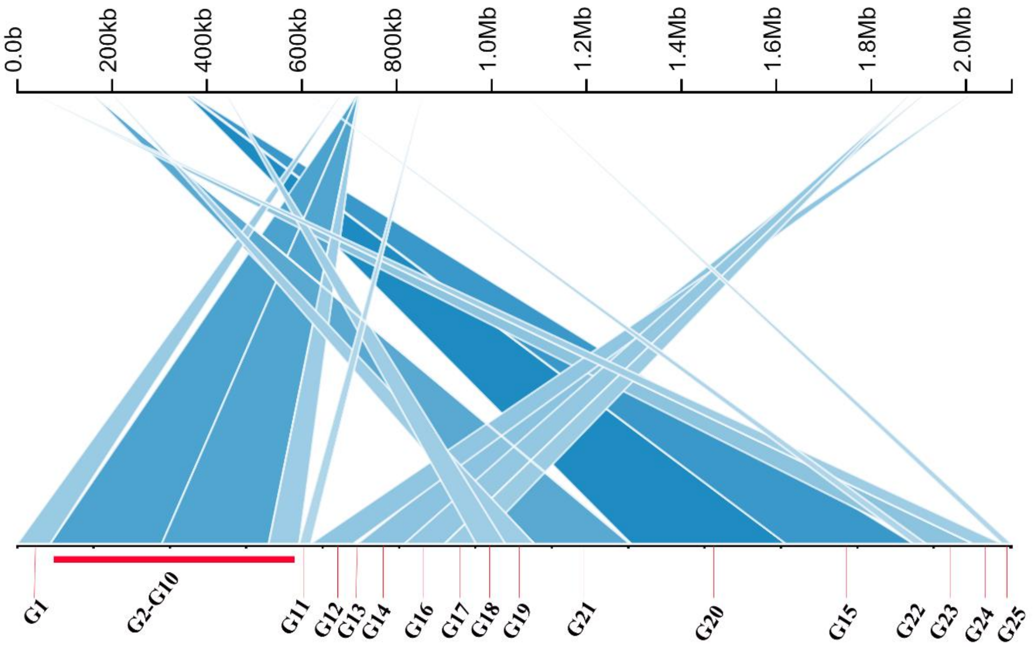

Appendix Figure S9. Twenty-five genes specific to HAC were found distributed in a variety location when they were mapped to the reference genome. G1-G10 were identified as contiguous genes, but they are not located in any MGE. The length of 20kb, 50kb, and 100kb flanking sequences of these contiguous genes were used for MGE prediction, respectively. MGEs were predicted with VRprofile (version 2.0). Prophages were predicted with Phage_Finder (version 2.1). ICEs were predicted with and ICEberg (version 1.0). Blastn program in NCBI also performed to see if they are annotated as MGEs.

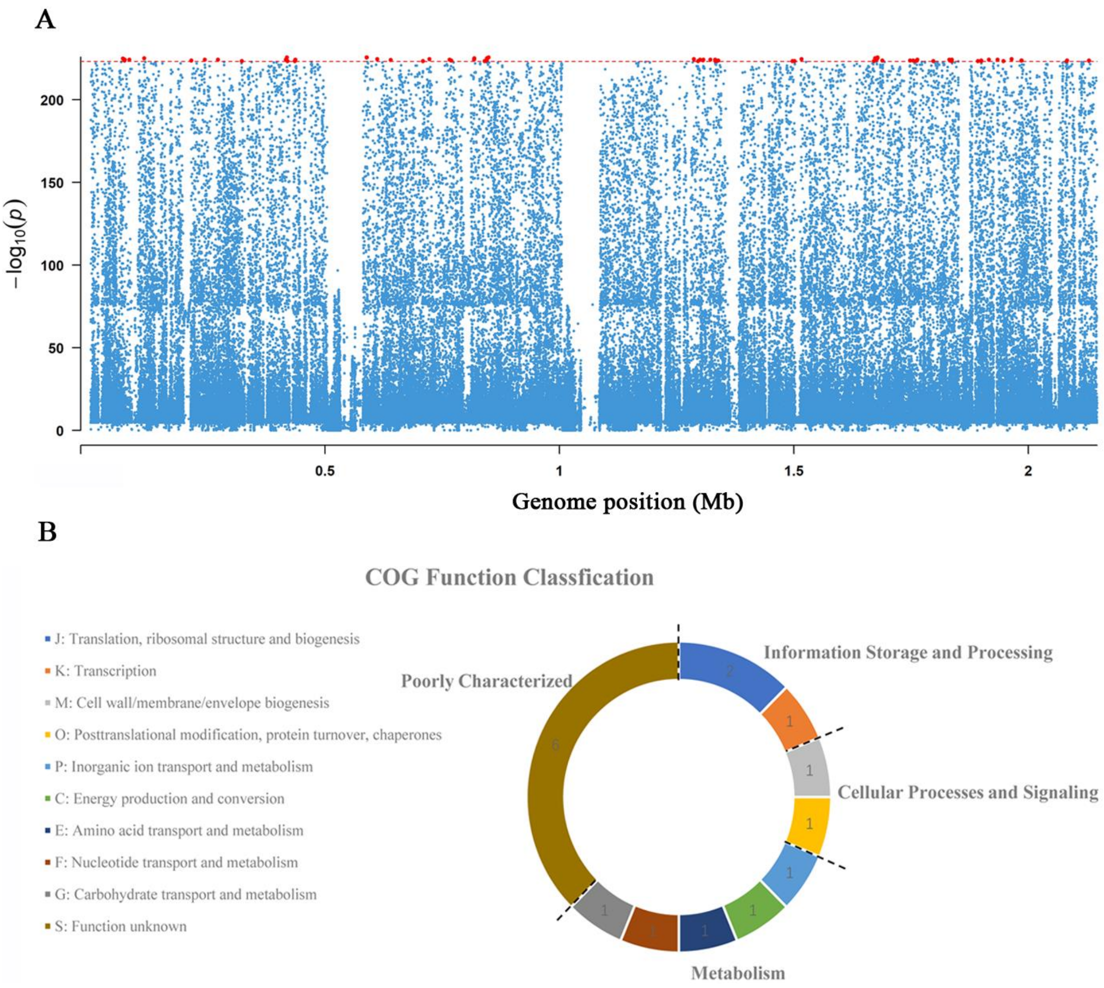

Appendix Figure S10. SNPs associated with human-associated isolates. A. show the statistical significance of the single-nucleotide polymorphisms (SNPs) using the same methods in Figure 3. The red line designates the genome-wide suggestive P-value thresholds while the variants highlighted in red were identified as significant SNPs by GEMMA method. B. COG functional classifications of 16 genes affected by missense variants.

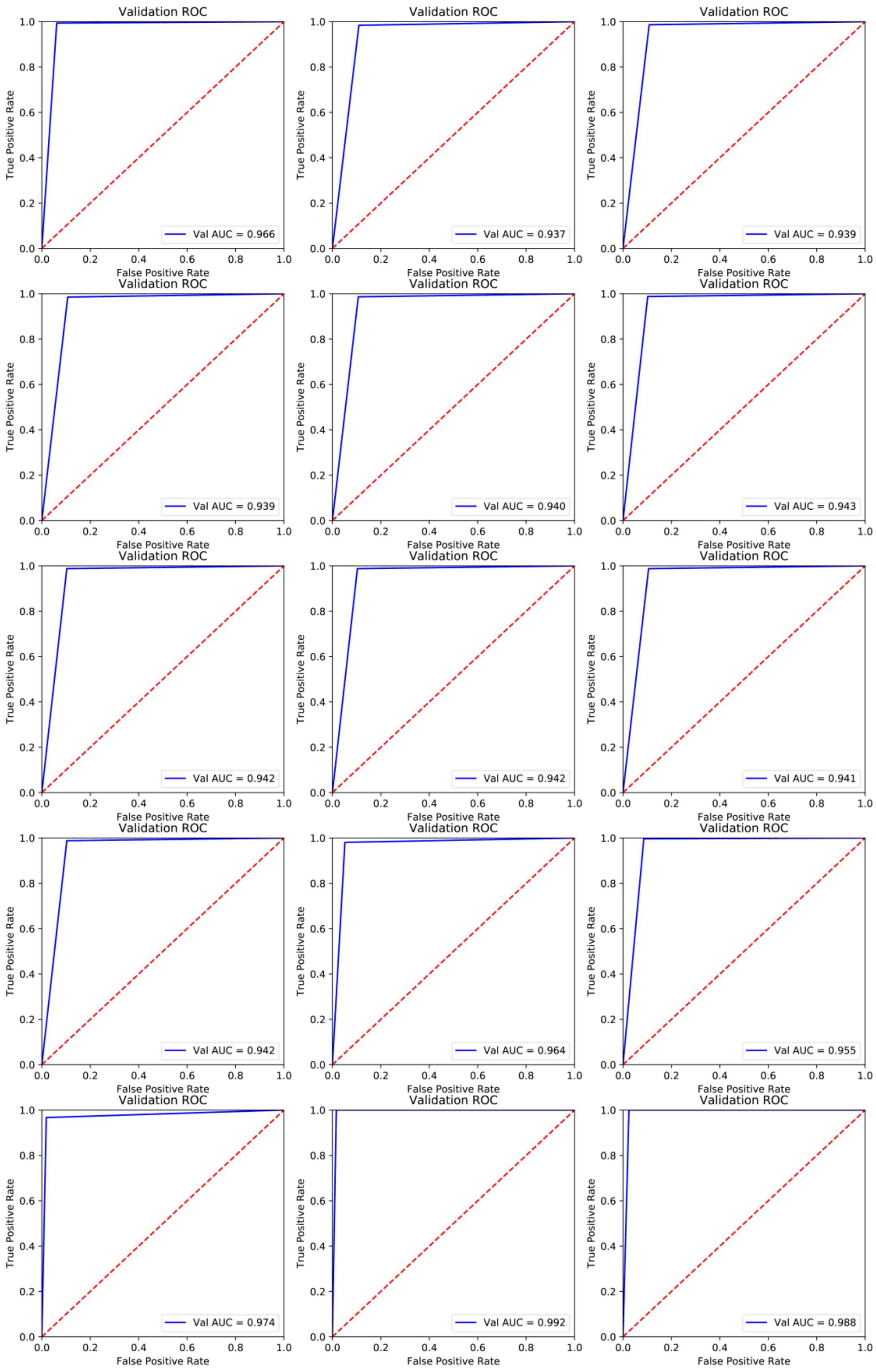

Appendix Figure S11. Continued.

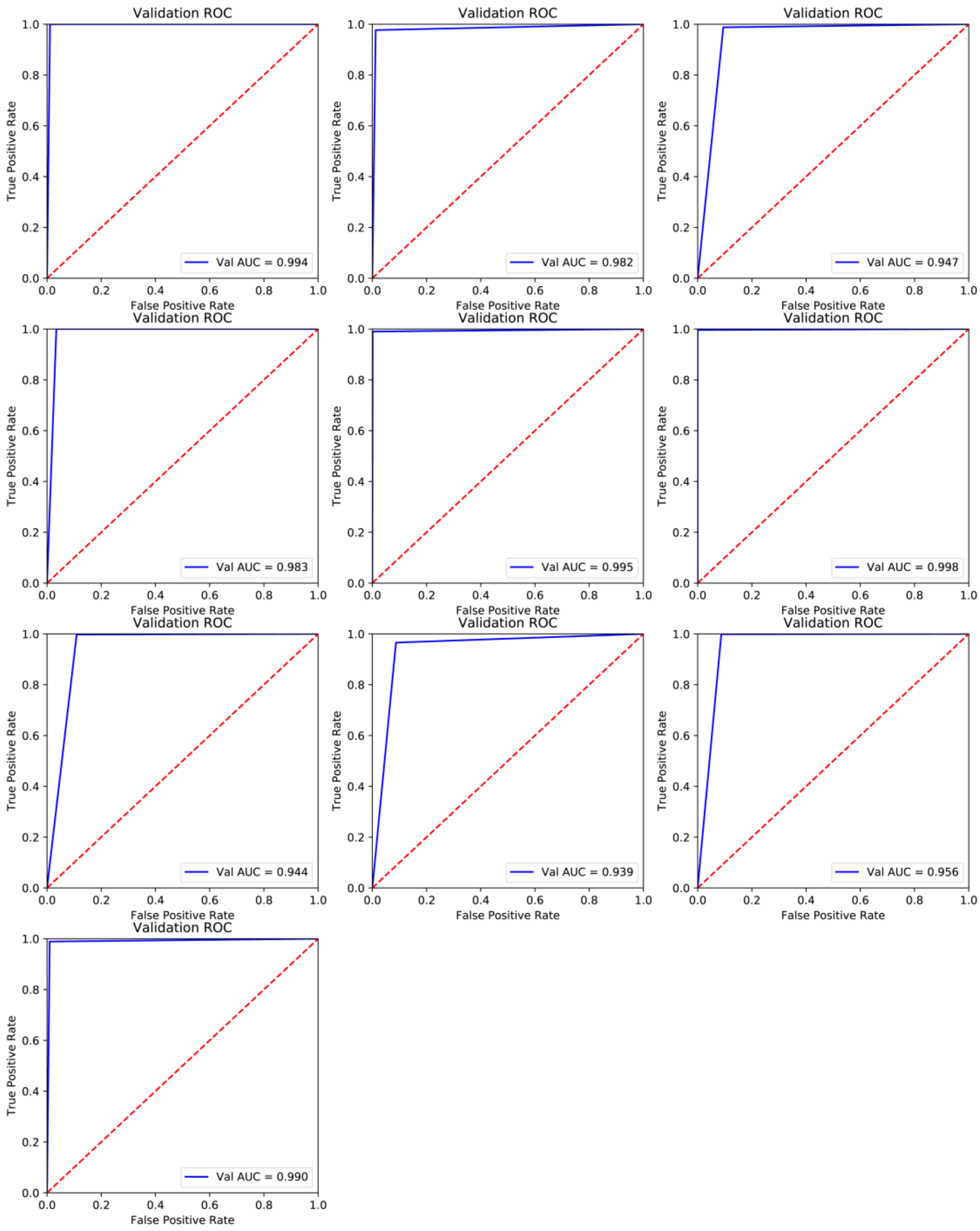

Appendix Figure S11. Receiver operating characteristic (ROC) curve analysis to assess the accuracy of 25 HAC-specific markers (area under the curve, AUC) in prediction of isolates from human-associated clade. Twenty-five predictive validity models with good predictive ability (AUC value >0.9) were established.

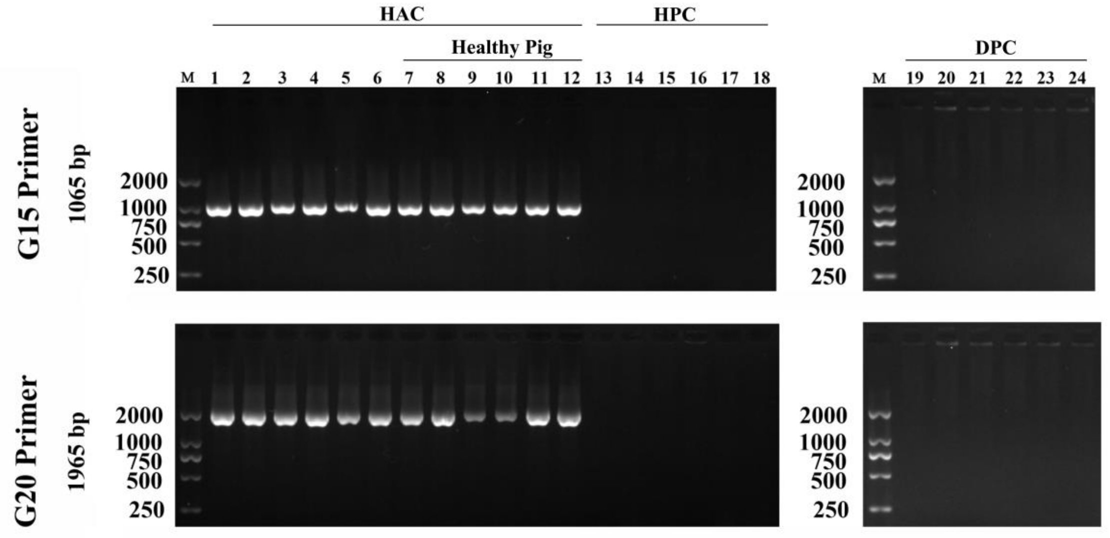

Appendix Figure S12. Agarose gel showing the expected amplicon sizes of two human-associated markers from 24 isolates in the training collection. Amplicons of size 1065bp and 1965bp correspond to the HAC-specific marker (G15 and G20), and were produced by all isolates in HAC. No PCR amplicons were observed with isolates of DPC and clade HPC. Lane M contains DNA ladder with sizes indicated on the left (bp).

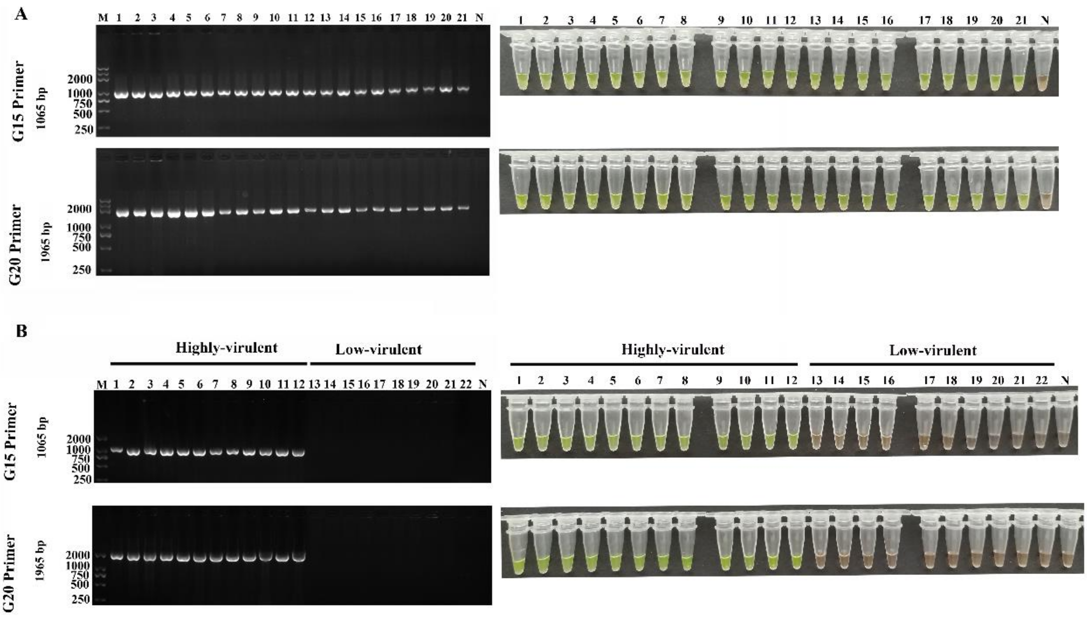

Appendix Figure S13. Development of PCR assays for the identification of human-associated and highly virulent *Streptococcus suis*. A. Twenty-one human isolates were collected from patients. B. Twenty-two isolates were taken from published research, of which 12 isolates (lane 1: human; lanes 2-10: diseased pigs; lanes11-12: healthy pigs) have been described as high-virulence and 10 isolates (lanes 13-15: diseased pigs; lanes 16-22: healthy pigs) have been described as low-virulence. All the primer pairs yield specific gene products and generate no nonspecific products as visualized by agarose gel electrophoresis and SYBR Green I in amplification tubes. The yellow green shade in tubes indicates a positive reaction, which can be easily distinguished from the orange shade that indicates negative reaction or no reaction in the negative control. Lane N = negative control (no DNA), and lane M = DL 2,000-bp DNA size marker.

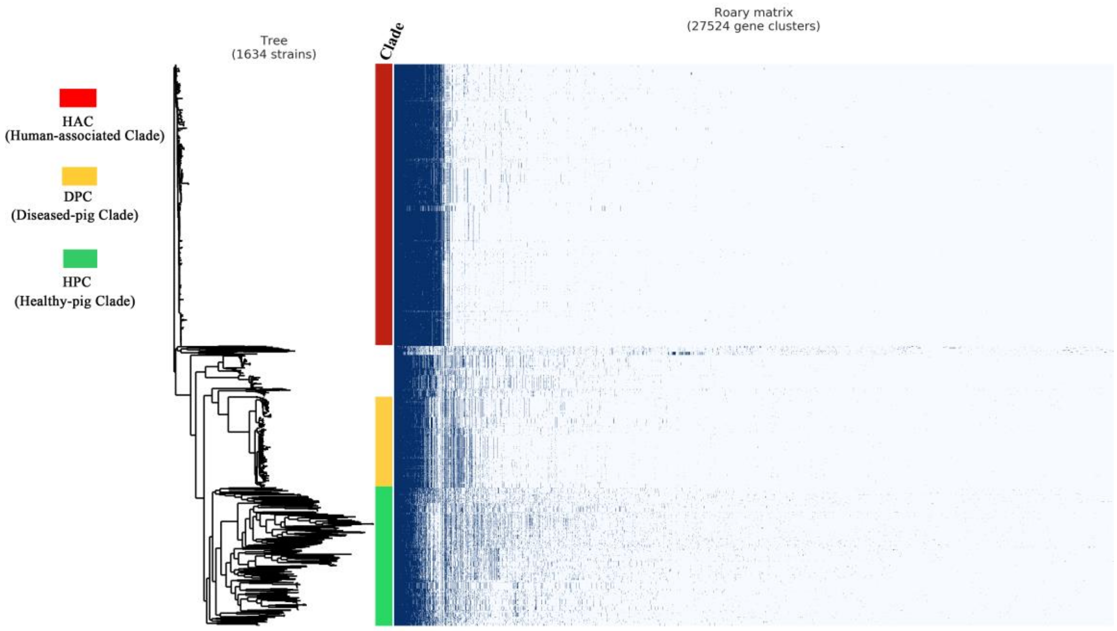

Appendix Figure S14. The isolates in HPC (Healthy-pig clade) had the larger pan-genome than those in HAC (Human-associated clade) and DPC (Diseased-pig clade). Heatmap showing the distribution of the pan genome among isolates. Dark blue indicates presence of a gene and light blue indicates absence of a gene. Each line represents an isolate. The isolates are vertically ordered based on similarity in a phylogenetic tree.

Appendix Table S1. Comparison of mortalities in zebrafish inoculated with twenty-five representative *S. suis* isolates from HAC (Human-associated Clade) and HPC (Healthy-pig Clade).

Clade	Host Source	Strains	Number of death/total	Group number of death/total	*P* -value[a] (Fisher's exact test, Dead vs Alive)
HAC (Human-associated Clade)	Healthy pig	1024-1	10/10	149/190	HAC VS HPC: P<0.05 (0.0001)<br>HAC VS SC19 : P>0.05 (0.8813)<br>HPC VS SC19: P< 0.05 (0.0341)
	Healthy pig	2283	8/10		
	Healthy pig	2355	9/10		
	Healthy pig	812	7/10		
	Healthy pig	832	7/10		
	Human	CH52	8/10		
	Human	CH61	10/10		
	Human	CH53	10/10		
	Human	LSM102	8/10		
	Human	CH39	7/10		
	Diseased pig	CPD17	12/20		
	Human	CH97	9/10		
	Human	CH65	16/20		
	Human	CH3	9/10		
SC19	Diseased pig	SC19	19/30	21/30	
HPC (Healthy-pig Clade)	Healthy pig	1247	4/10	38/110	
	Healthy pig	13	4/10		
	Healthy pig	1783	2/10		
	Healthy pig	1053	5/10		
	Healthy pig	3096	5/10		
	Healthy pig	262	7/20		
	Healthy pig	142	4/10		
	Healthy pig	1047	3/10		
	Healthy pig	1214	4/10		
	Healthy pig	1012	0/10		

The cumulative mortalities were calculated using the combined data from representative isolates in each clade. a, Fisher's exact test was used to caculate Dead vs Alive at the end of study. *P* < 0.05 was considered significant.

Appendix Table S2. Genes identified special for lineage III.

Gene code	Gene/orthogroup	Annotation code	Functional annotation	
G1	group_842	CLLDIKEE_01019	DNA processing protein DprA	Replication, recombination and repair
G2	group_844	DFLLMFLL_00686	Maltose O-acetyltransferase	Amino acid transport and metabolism
G3	group_1252	PMGPKGND_01345	hypothetical protein	Function unknown
G4	tetO	PMGPKGND_01346	Tetracycline resistance protein TetO	Signal transduction mechanisms
G5	group_9	PMGPKGND_01344	helicase	Replication, recombination and repair
G6	group_270	OJMOKAOK_01442	hypothetical protein	Function unknown
G7	group_21	PMGPKGND_01341	Agglutinin receptor	Function unknown
G8	group_2465	OJMOKAOK_01461	DNA repair protein RadA domain	Function unknown
G9	group_289	PMGPKGND_01342	hypothetical protein	Function unknown
G10	group_3510	OJMOKAOK_01463	RNA polymerase sigma factor, sigma-70 family	Function unknown
G11	pre	OJMOKAOK_01460	Plasmid recombination enzyme	Replication, recombination and repair
G12	group_1577	OJMOKAOK_01458	hypothetical protein	Function unknown
G13	spo0C	OJMOKAOK_01459	Chromosome-partitioning protein Spo0J	Transcription
G14	group_2036	OJMOKAOK_01464	sigma-70, region 4	Function unknown
G15	group_4023	OJMOKAOK_01462	sigma-70, region 4	Function unknown
G16	group_408	FBHLMCBI_00427	Resolvase	Replication, recombination and repair
G17	group_4022	OJMOKAOK_01457	hypothetical protein	Function unknown
G18	group_4016	OJMOKAOK_01434	Bacterial mobilization protein (MobC)	Function unknown
G19	group_4017	OJMOKAOK_01435	hypothetical protein	Function unknown
G20	group_3509	OJMOKAOK_01433	relaxase mobilization nuclease domain protein	Intracellular trafficking, secretion, and vesicular transport
G21	regX3	OJMOKAOK_01431	Sensory transduction protein regX3	Signal transduction mechanisms
G22	group_3508	OJMOKAOK_01429	putative ABC transporter ATP-binding protein YxlF	Defense mechanisms
G23	nisI_1	OJMOKAOK_01426	Nisin immunity protein	Function unknown
G24	group_4015	OJMOKAOK_01428	ABC transporter, permease	Function unknown
G25	nisP	OJMOKAOK_01432	Nisin leader peptide-processing serine protease NisP	Post-translational modification, protein turnover, and chaperones
G26	group_3503	OJMOKAOK_01425	sigma-70, region 4	Function unknown
G27	group_3507	OJMOKAOK_01427	hypothetical protein	Function unknown
G28	group_2463	OJMOKAOK_01424	Site-specific recombinase	Replication, recombination and repair
G29	group_12	OJMOKAOK_01448	helicase	Replication, recombination and repair
G30	nisC	FBHLMCBI_00452	Nisin biosynthesis protein NisC	Function unknown

G31	group_1072	FBHLMCBI_01912	hypothetical protein	Function unknown
G32	group_419	FBHLMCBI_00438	atpase involved in dna repair	Replication, recombination and repair
G33	group_837	FBHLMCBI_01916	atpase involved in dna repair	Replication, recombination and repair
G34	creC	OJMOKAOK_01430	Sensor protein CreC	Signal transduction mechanisms
G35	spxA_3	FBHLMCBI_01915	hypothetical protein	Function unknown
G36	group_335	OJMOKAOK_01447	hypothetical protein	Function unknown
G37	group_123	FBHLMCBI_01917	hypothetical protein	Function unknown
G38	group_620	FBHLMCBI_01922	hypothetical protein	Function unknown
G39	cmoB	FBHLMCBI_01923	traE protein	Intracellular trafficking, secretion, and vesicular transport
G40	group_125	CLLDIKEE_01014	Replication initiator protein A	Replication, recombination and repair
G41	group_328	FBHLMCBI_00436	Antitoxin PezA	Transcription
G42	ltxB	FBHLMCBI_00451	putative ABC transporter ATP-binding protein	Defense mechanisms
G43	group_191	FBHLMCBI_01919	Protein VirD4	Intracellular trafficking, secretion, and vesicular transport
G44	group_115	FBHLMCBI_00432	DNA primase	Replication, recombination and repair
G45	group_3520	FBHLMCBI_00430	hypothetical protein	Function unknown
G46	nsuA	FBHLMCBI_00449	Lantibiotic nisin-U	Function unknown
G47	group_537	FBHLMCBI_00437	Toxin PezT	Function unknown
G48	group_3523	FBHLMCBI_01925	Abi-like protein	Function unknown
G49	nisB	FBHLMCBI_00450	Nisin biosynthesis protein NisB	Function unknown
G50	group_983	FBHLMCBI_01920	hypothetical protein	Function unknown
G51	group_350	FBHLMCBI_01921	conjugative transposon membrane protein	Function unknown
G52	group_293	FBHLMCBI_01918	hypothetical protein	Function unknown
G53	group_128	PMGPKGND_01343	hypothetical protein	Function unknown
G54	group_175	DKFDCKMA_01474	Abortive infection protein AbiGII	Function unknown
G55	group_1063	FBHLMCBI_00442	hypothetical protein	Function unknown
G56	group_1201	FBHLMCBI_00287	Membrane	Function unknown
G57	group_1854	FBHLMCBI_01257	integral membrane protein	Function unknown
G58	xylB	FBHLMCBI_00809	Xylulose kinase	Carbohydrate transport and metabolism
G59	sorC_2	FBHLMCBI_00813	PTS system sorbose-specific EIIC component	Carbohydrate transport and metabolism
G60	xylA	FBHLMCBI_00808	Xylose isomerase	Carbohydrate transport and metabolism
G61	group_4050	FBHLMCBI_00810	Alpha-xylosidase BoGH31A	Carbohydrate transport and metabolism
G62	group_4051	FBHLMCBI_00811	Alpha-xylosidase BoGH31A	Carbohydrate transport and metabolism

G63	sorB_1	FBHLMCBI_00812	PTS system sorbose-specific EIIB component	Carbohydrate transport and metabolism
G64	manZ_2	FBHLMCBI_00814	PTS system mannose-specific EIID component	Carbohydrate transport and metabolism
G65	group_4054	FBHLMCBI_00815	PTS system fructose IIA component	Carbohydrate transport and metabolism
G66	nagC	FBHLMCBI_00807	N-acetylglucosamine repressor	Transcription
G67	group_3522	FBHLMCBI_00816	hypothetical protein	Function unknown
G68	group_746	FBHLMCBI_01259	Transposase	Replication, recombination and repair
G69	group_1199	FBHLMCBI_01253	Membrane	Function unknown
G70	polA_2	FBHLMCBI_01254	DNA polymerase I	Replication, recombination and repair
G71	group_2978	FBHLMCBI_00418	Major Facilitator Superfamily	Carbohydrate transport and metabolism

Appendix Table S3. Genes identified special for lineage II.

Gene code	Gene/orthogroup	Annotation code	Functional annotation	
G1	polA	CLLDIKEE_01387	DNA polymerase I	Replication, recombination and repair
G2	group_1855	CLLDIKEE_01384	hypothetical protein	Function unknown
G3	group_1200	CLLDIKEE_01388	hypothetical protein	Function unknown
G4	group_91	CLLDIKEE_01380	Avirulence protein AvrXa10	Cell wall/membrane/envelope biogenesis
G5	group_2660	CLLDIKEE_00654	hypothetical protein	Function unknown
G6	group_179	CLLDIKEE_01003	traE protein	Intracellular trafficking, secretion, and vesicular transport
G7	pezT	CLLDIKEE_00965	Toxin PezT	Function unknown
G8	pezA_1	CLLDIKEE_00966	Antitoxin PezA	Transcription
G9	maa	CLLDIKEE_01021	Maltose O-acetyltransferase	Amino acid transport and metabolism
G10	group_348	CLLDIKEE_01005	hypothetical protein	Function unknown
G11	dprA	CLLDIKEE_01020	DNA processing protein DprA	Replication, recombination and repair
G12	xerD_1	CLLDIKEE_00928	Tyrosine recombinase XerD	Replication, recombination and repair
G13	tetD	CLLDIKEE_00925	Transposon Tn10 TetD protein	Transcription
G14	group_3874	CLLDIKEE_00929	hypothetical protein	Function unknown
G15	tetM	CLLDIKEE_00950	Tetracycline resistance protein TetM from transposon TnFO1	Signal transduction mechanisms/ Translation, ribosomal structure and biogenesis
G16	group_1787	CLLDIKEE_00951	conjugative transposon protein	Replication, recombination and repair
G17	iap	CLLDIKEE_00952	putative endopeptidase p60	Cell wall/membrane/envelope biogenesis
G18	group_1793	CLLDIKEE_00953	conjugative transposon membrane protein	Replication, recombination and repair
G19	dnaG_1	CLLDIKEE_00970	DNA primase	Replication, recombination and repair
G20	group_3287	CLLDIKEE_00949	DNA-binding helix-turn-helix protein	Transcription
G21	group_1286	CLLDIKEE_00954	Conjugal transfer protein	Intracellular trafficking, secretion, and vesicular transport
G22	group_3285	CLLDIKEE_00948	sigma-70, region 4	Function unknown
G23	phoR	CLLDIKEE_00931	Phosphate regulon sensor protein PhoR	Signal transduction mechanisms
G24	group_2241	CLLDIKEE_00933	Abc transporter integral membrane protein	Function unknown
G25	arlR_1	CLLDIKEE_00932	Response regulator ArlR	Signal transduction mechanisms
G26	group_2707	CLLDIKEE_00941	relaxase mobilization nuclease domain protein	Intracellular trafficking, secretion, and vesicular transport
G27	group_2708	CLLDIKEE_00943	Bacterial mobilization protein (MobC)	Function unknown
G28	group_3290	CLLDIKEE_00955	conjugative transposon membrane protein	Replication, recombination and repair
G29	yxlF_2	CLLDIKEE_00934	putative ABC transporter ATP-binding protein YxlF	Inorganic ion transport and metabolism/Defense mechanisms
G30	group_2709	CLLDIKEE_00944	Divergent AAA domain protein	Function unknown
G31	group_3965	AABLGPLK_00184	hypothetical protein	Function unknown
G32	group_2711	CLLDIKEE_00946	Excisionase	Replication, recombination and repair
G33	bmrA_1	CLLDIKEE_00935	Multidrug resistance ABC transporter ATP-binding/permease protein BmrA	Defense mechanisms
G34	recF_1	CLLDIKEE_00964	DNA replication and repair protein RecF	Replication, recombination and repair
G35	group_271	CLLDIKEE_00968	hypothetical protein	Function unknown
G36	degU	CLLDIKEE_00972	Transcriptional regulatory protein DegU	Signal transduction mechanisms
G37	group_3284	CLLDIKEE_00942	Bacterial mobilization protein (MobC)	Function unknown
G38	group_3885	CLLDIKEE_00994	hypothetical protein	Function unknown
G39	group_332	CLLDIKEE_00995	hypothetical protein	Function unknown
G40	immR_1	CLLDIKEE_00930	HTH-type transcriptional regulator ImmR	Transcription
G41	liaS_1	CLLDIKEE_00973	Sensor histidine kinase LiaS	Signal transduction mechanisms
G42	group_2244	CLLDIKEE_00940	hypothetical protein	Function unknown
G43	group_133	CLLDIKEE_00997	single-strand binding family protein	Function unknown
G44	group_1785	CLLDIKEE_00945	Transposase from transposon Tn916	Replication, recombination and repair
G45	group_3880	CLLDIKEE_00947	conjugative transposon protein	Replication, recombination and repair
G46	group_286	CLLDIKEE_00998	LamG domain protein jellyroll fold domain protein	Function unknown

Appendix Table S4. Summary of the genome-wide significant and suggestive accessory genes associated with human-associated isolates identified by the GWAS analysis.

Gene code	Gene/orthogroup	Annotation code	Functional annotation	Localization Class
G1	group_10414	DNCANAHA_00665	hypothetical protein	secreted
G2	nisI	DNCANAHA_00194	Nisin immunity protein	secreted
G3	group_9278	DNCANAHA_00011	hypothetical protein	cytoplasm
G4	group_17264	DNCANAHA_00049	Energy-coupling factor transporter tra	inner membrane
G5	group_10366	DNCANAHA_00013	hypothetical protein	cytoplasm
G6	group_18663	DNCANAHA_00048	hypothetical protein	inner membrane
G7	crp	DNCANAHA_00046	CRP-like cAMP-activated global tran	cytoplasm
G8	group_14293	DNCANAHA_01455	hypothetical protein	secreted
G9	rizA	DNCANAHA_00196	L-arginine-specific L-amino acid liga	cytoplasm
G10	group_17279	DNCANAHA_01020	hypothetical protein	cytoplasm
G11	udp	DNCANAHA_00047	Uridine phosphorylase	cytoplasm
G12	group_17296	DNCANAHA_01833	hypothetical protein	cytoplasm
G13	group_1166	DNCANAHA_00457	hypothetical protein	cytoplasm
G14	group_14299	DNCANAHA_01600	hypothetical protein	secreted
G15	kcsA_1	DNCANAHA_01682	pH-gated potassium channel KcsA	inner membrane
G16	thiM	DNCANAHA_00044	Hydroxyethylthiazole kinase	secreted
G17	thiD	DNCANAHA_00043	Hydroxymethylpyrimidine/phosphom	cytoplasm
G18	group_9612	DNCANAHA_01832	hypothetical protein	cytoplasm
G19	group_12805	DNCANAHA_00068	hypothetical protein	cytoplasm
G20	corA	DNCANAHA_00738	Magnesium transport protein CorA	inner membrane
G21	ppaX	DNCANAHA_01491	putative protein	cytoplasm
G22	group_2911	DNCANAHA_00466	hypothetical protein	inner membrane
G23	ecfA2_2	DNCANAHA_00051	Energy-coupling factor transporter A	inner membrane
G24	group_734	DNCANAHA_01050	hypothetical protein	secreted
G25	group_11497	DNCANAHA_00012	hypothetical protein	cytoplasm
G26	nadR_2	DNCANAHA_00585	Trifunctional NAD biosynthesis/regu	cytoplasm
G27	group_1732	DNCANAHA_00016	hypothetical protein	cytoplasm
G28	group_12834	DNCANAHA_00587	Bifunctional NMN adenylyltransferas	cytoplasm
G29	group_6991	DNCANAHA_00010	Type IIS restriction enzyme Eco57I	cytoplasm
G30	copA_3	DNCANAHA_01015	Copper-exporting P-type ATPase	inner membrane
G31	group_13791	DNCANAHA_01293	hypothetical protein	cytoplasm
G32	group_14889	DNCANAHA_01294	hypothetical protein	cytoplasm
G33	hxpB	DNCANAHA_00042	Phosphorylated carbohydrates phosph	cytoplasm
G34	group_20858	DNCANAHA_01408	hypothetical protein	secreted
G35	thiE	DNCANAHA_00045	Thiamine-phosphate synthase	cytoplasm
G36	pnuC	DNCANAHA_00586	Nicotinamide riboside transporter Pn	inner membrane
G37	albA	DNCANAHA_00560	Antilisterial bacteriocin subtilosin bio	cytoplasm
G38	group_8383	DNCANAHA_00514	hypothetical protein	secreted
G39	group_13837	DNCANAHA_01976	hypothetical protein	cytoplasm
G40	group_17275	DNCANAHA_00591	hypothetical protein	inner membrane
G41	nagH	DNCANAHA_00462	hypothetical protein	secreted
G42	group_5015	DNCANAHA_01403	Hyaluronate lyase	secreted
G43	group_8928	DNCANAHA_01889	hypothetical protein	cytoplasm
G44	group_6943	IHDAOOLN_01662	hypothetical protein	inner membrane
G45	group_20844	DNCANAHA_00557	hypothetical protein	inner membrane
G46	group_9228	DNCANAHA_01292	hypothetical protein	secreted
G47	group_20845	DNCANAHA_00559	hypothetical protein	secreted
G48	uup	DNCANAHA_01183	ABC transporter ATP-binding protei	inner membrane
G49	group_1928	DNCANAHA_00465	hypothetical protein	fimbrium
G50	group_3039	DNCANAHA_00656	hypothetical protein	cytoplasm
G51	group_9183	DNCANAHA_00661	hypothetical protein	inner membrane
G52	group_17277	DNCANAHA_00627	hypothetical protein	secreted
G53	group_9791	DNCANAHA_00628	hypothetical protein	cytoplasm
G54	group_17276	DNCANAHA_00620	hypothetical protein	secreted
G55	group_1584	DNCANAHA_01884	hypothetical protein	secreted
G56	group_4958	DNCANAHA_00463	Hyaluronoglucosaminidase	secreted

G57	group_4333	DNCANAHA_00674	Internalin-A	secreted
G58	group_4339	DNCANAHA_00676	hypothetical protein	inner membrane
G59	group_7301	DNCANAHA_01288	hypothetical protein	cytoplasm
G60	group_11927	DNCANAHA_00625	PTS system EIIBC component	inner membrane
G61	group_1454	DNCANAHA_00037	hypothetical protein	cytoplasm
G62	group_9764	DNCANAHA_00181	hypothetical protein	cytoplasm
G63	group_1587	IHDAOOLN_01145	Trifunctional NAD biosynthesis/regu	cytoplasm
G64	group_13309	IHDAOOLN_01571	Magnesium transport protein CorA	inner membrane
G65	group_5648	IHDAOOLN_02052	putative protein	cytoplasm
G66	group_1397	IHDAOOLN_00715	Nisin immunity protein	secreted

Note: All 25 accessory genes specific to HAC identified with chi-square test were indicated with gray background.

Appendix Table S5. Genes identified special for human-associated clade by Chi-square analysis.

Gene code	Gene/orthogroup	Annotation code	Functional annotation	
G1	group_11497	DNCANAHA_00012	hypothetical protein	Function unknown
G2	hxpB	DNCANAHA_00042	Phosphorylated carbohydrates phosphatase/Hydrolase	Coenzyme transport and metabolism
G3	thiD	DNCANAHA_00043	Hydroxymethylpyrimidine/phosphomethylpyrimidine kinase	Coenzyme transport and metabolism
G4	thiM	DNCANAHA_00044	Hydroxyethylthiazole kinase	Coenzyme transport and metabolism
G5	thiE	DNCANAHA_00045	Thiamine-phosphate synthase	Coenzyme transport and metabolism
G6	crp	DNCANAHA_00046	CRP-like cAMP-activated global transcriptional regulator/Crp Fnr family	Signal transduction mechanisms
G7	udp	DNCANAHA_00047	Uridine phosphorylase	Nucleotide transport and metabolism
G8	group_18663	DNCANAHA_00048	Signal transduction histidine kinase, lyts	Function unknown
G9	group_17264	DNCANAHA_00049	Energy-coupling factor transporter transmembrane protein EcfT/Cobalt transport protein	Inorganic ion transport and metabolism
G10	ecfA2_2	DNCANAHA_00051	Energy-coupling factor transporter ATP-binding protein EcfA2/ABC transporter	Inorganic ion transport and metabolism
G11	group_9764	DNCANAHA_00181	Abortive infection bacteriophage resistance protein	Defense mechanisms
G12	group_1928	DNCANAHA_00465	Lpxtg-motif cell wall anchor domain protein	Cell wall/membrane/envelope biogenesis
G13	group_20845	DNCANAHA_00559	hypothetical protein	Function unknown
G14	albA	DNCANAHA_00560	Antilisterial bacteriocin subtilosin biosynthesis protein AlbA/Pyrroloquinoline quinone biosynthesis protein E	Replication, recombination and repair
G15	group_9228	DNCANAHA_01292	ATP-binding protein	Function unknown
G16	nadR_2	DNCANAHA_00585	Trifunctional NAD biosynthesis/regulator protein NadR/Nicotinamide-nucleotide adenylyltransferase	Transcription/**Virulence factor**
G17	pnuC	DNCANAHA_00586	Nicotinamide riboside transporter PnuC	Coenzyme transport and metabolism/**Virulence factor**
G18	group_9183	DNCANAHA_00661	hypothetical protein	Function unknown
G19	group_17279	DNCANAHA_01020	hypothetical protein	Function unknown
G20	group_7301	DNCANAHA_01288	hypothetical protein	Function unknown
G21	group_734	DNCANAHA_01050	hypothetical protein	Function unknown
G22	group_14299	DNCANAHA_01600	hypothetical protein	Function unknown
G23	group_9612	DNCANAHA_01832	hypothetical protein	Function unknown
G24	group_17296	DNCANAHA_01833	hypothetical protein	Function unknown
G25	group_13837	DNCANAHA_01976	hypothetical protein	Function unknown

G15 and G20 were selected as diagnostic markers specific for human-associated *S. suis* .

Appendix Table S6. Summary of genes affected by 17 missense variants that correlate with human-associated isolates.

POS	REF/ALT	Annotation	Gene_ID	Protein Name
rs72180	T/C	missense_variant	SSUBM407_RS00405	30S ribosomal protein S10
rs243228	T/C	missense_variant	SSUBM407_RS01265	dihydroorotate oxidase
rs418761	A/G	missense_variant	SSUBM407_RS02060	Cof-type HAD-IIB family hydrolase
rs419769	T/C	missense_variant	SSUBM407_RS02060	Cof-type HAD-IIB family hydrolase
rs436696	T/C	missense_variant	SSUBM407_RS02195	beta-galactosidase
rs611132	C/T	missense_variant	SSUBM407_RS03030	ferrous iron transport protein B
rs722184	G/A	missense_variant	SSUBM407_RS03520	homoserine O-succinyltransferase
rs817247	A/G	missense_variant	SSUBM407_RS03975	aconitate hydratase AcnA
rs839952	C/T	missense_variant	SSUBM407_RS04065	TIGR03943 family protein
rs1286512	G/A	missense_variant	SSUBM407_RS06310	hypothetical protein
rs1299547	C/T	missense_variant	SSUBM407_RS06385	CBS domain-containing protein
rs1500939	A/G	missense_variant	SSUBM407_RS07330	DJ-1/PfpI family protein
rs1516052	C/T	missense_variant	SSUBM407_RS07440	bifunctional oligoribonuclease/PAP phosphatase NrnA
rs1672817	A/G	missense_variant	SSUBM407_RS08295	transcription elongation factor GreA
rs1676399	A/T	missense_variant	SSUBM407_RS08310	UDP-N-acetylmuramate--L-alanine ligase
rs1933882	T/C	missense_variant	SSUBM407_RS09475	elongation factor Ts
rs2128884	A/G	missense_variant	SSUBM407_RS10380	helix-turn-helix domain-containing protein

Appendix Table S7. Genes identified special for diseased-pig clade.

Gene code	Gene/orthogroup	Annotation code	Functional annotation	
G26	group_18729	JDODEKPG_00059	DUF4268 domain-containing protein	Function unknown
G27	group_16363	JDODEKPG_00186	aromatic acid exporter family protein	Function unknown
G28	group_16364	JDODEKPG_00254	DUF1310 family protein	Function unknown
G29	group_8214	JDODEKPG_00686	Putative DNA repair helicase RadD	Replication, recombination and repair
G30	nudG_2	JDODEKPG_00687	CTP pyrophosphohydrolase	Replication, recombination and repair
G31	cas1	JDODEKPG_00926	CRISPR-associated endonuclease Cas1	Replication, recombination and repair
G32	cas2	JDODEKPG_00927	CRISPR-associated endoribonuclease Cas2	Replication, recombination and repair
G33	group_17366	JDODEKPG_01920	hypothetical protein	Function unknown
G34	group_15626	JDODEKPG_01921	hypothetical protein	Function unknown
G35	group_17367	JDODEKPG_01922	hypothetical protein	Function unknown

Appendix Table S8. PCR primers were designed for human-associated *S. suis* .

Clade	Gene	Primer name	Primer(5'-3')	Product length(bp)
HAC (Human-associated clade)	group_9228	Primer_1_F	AAAGAGGAGGTTGGAAGAGGTAG	1065
		Primer_1_R	CTGAGGAAAATTGAGAGCATAGG	
	group_7301	Primer_2_F	TGACAAGGTATTTGGGTGGGATG	1965
		Primer_2_R	AAGAAGGTCGTAGTTCTGGGAGC	

The target region of two pairs of primers.

>Primer 1

AAAGAGGAGGTTGGAAGAGGTAGCCTGCCTTGTTTTGAGGACTATATTCCCTACTCAGTAGCAACAGCTTCTAATATTCC
AACGGAGAAATCAGAGAAGTTCATCAGTCAGACAATCGAAAAATTATTGGATGGCATTGTACCTGAAAACAAAGAACA
AGAATACATTCTTGTCCTCCTGGCAACGCCAGTTTTGGATGTTACAGATAGAAAATTGCATCTTTCTCAGATTTATTCTAA
CCTATCTCCTTATGCCACTTGGCAGACCCAGTTCCAATTGACAGAATCAGATTCCAGAGGCTCTTCTGCAACTGTTGGGG
TTAATATTGGTGCCAGTGTCGGAAGCCAGACAGGTCACAATCTAACCCAAACCCATTCGCAAGGTCAAACTGAGTCAAG
AGGAACTAGTGAGACGGAGACCAATACCAAAGGAAGTGGTTGGCAAGCAGGTGTAAATATTAAAATACTCAACGGAGG
TCATAATTGGAACAAAAGCAAATCAATCGCCAAAGGCATCACCAATACAATAGGCAAAACAGCCTCGGTTGCCAATGCC
GTTGGGAAAACAGTTTCTAGCAGTCTAGGTGCCAATTTTGGAGCTAATTTTGCTCGAACTTCCAGTGTGACTGCTACCATT
GGGAAAAATGAAGGCATTAGTCAGTCCTTTGTCAACCACCATGTTCAGCACGCTCTAAAAAATTTAGACAAGCAGATGG
AGCGTTTGGAATTGTCTACTGCCTTGGGACTATGGGATTTTTCTGCCTATGTTTTGAGTGAAGATCCAACTATTGCCAATA
ACGTTGCCCATTCCTATTTGGCCTTGACTCAGGGAGAAGAATCGCATCTGTCGCAAACAGTTGTCAATCTTTGGCGTGGG
GATGTTCAAATGGAACGTGATAAGGCTAAAGCACTGATTGCCTACTTGAGAGATTTACGCCACCCTCTCTTTGGTTTCAA
CCCAGAAATTCTAGAGATTGATGAAGAATTTGCAGTTTATCCAGAAATCGTCACAGCAGCCACGCCACTGTCTGGAAAG
GAATTAGCCTATGCTCTCAATTTTCCTCAG

>Primer 2

TGACAAGGTATTTGGGTGGGATGCCAATACCGTATTTAGCTGGTTATTTTTGATTCTTCTTTTTATTGGCTCCCTTACAAC
GATTTGGTATATAGTTGAAAATAAGACGATAAAATCATTGACAATTGGTGGCAAAAATGTTTCATCAGAGATTGATATTT
TTTCTGATCAGTCGATGAGAGTTTCCTATTTTGACAAATACCTAGATGATGTGTTGTATCTTTTGAATGAATCAAGAGCAG
ATGTCATTGTTTTTGAGGATATCGACCGTTTTGAGAATAACACTATTTTTGCCAAGATTAAGGAACTAAACGTATTAGTC
AACAATAAGCGAAAAATAGCTAAGAAATCATCAAAGCTAGTGTTTCTTTACCTGATACGAGATGATTTATTCATTTCAAA
AGAAAGAACTAAATTTTTTGATTTTATCATCCCAGTCCTTCCAGTTATTACGAGTTCAAACTCTAGCGATAAGTTGACAAC
TACTTTAAAAGAAATGGGAATAAAATCAGGATTAGCGGATGACTTCCTCTTCAGAATCTCGCTTTATATAGATGATATGA
GATTGCTTAATAATATCTGTAATGAATTTTATTCTTATCAGCTTGAATTGACACATGACAAGACGGGTGAGAAAAATGCA
TTGGATCTTGATTTGAAAAAAATATTTGCAATGATTGTCTACAAAAATATTTTCCCGAAAGACTTTTCTGAGTTGCAGAAT
AATCAAGGTTTTCTATATTCATTGTTTAATGAGAAAGAAGTTAGAAGAAGTGAAAAACTTACGAAGATTGATAGTGAAA
AACTGCATTTAGAGAATAAACTGAGAAAGATTCAGAGTGAGCACATTCAAGATGAAATTGAACTCTATGGAACAATTTT
TAAAATTCCAAATGGGAGAAAGGTTGTCAGTGTCAATGACAAATTTCAAGATGAGTTTACTTCTTATCATGATTTTATTTC
TGAAATGCTGGTGGAAGGAAGTCGTATCATCTCCTATGTGGATTTTGATGATGCTTACCGTAATGTAGGTATTAAAACAG
AAAATATGGATTCAATTTTTCCAGAGAAAGATTCTCCAGAGTTTAAGGAACGCCTTGATACTGTAAGAAGTCGCAATAAT
AGTAAAGAGTTGCAACAAAAAATTGCTTCATTAAATCAAAAACGGAGCTTTGTTGAAAAACAATTGATTGCGGACATCT
ATACTAATCAAGAAATACATGAATTTGCTAAGAGTAGAGAAGAATTTATTGATATAGAAAAAAATCAGCAATTTGATAT
TATTTACTTCTTATTGAAAAACTCTTATATAGATGAAACCTATCCAGATTATTTGACCTATTTTTATGGCAATGTCTTAACT
AAGAATGATAAAGAATTTTTGAGAAATATTTCCAGTGGAAGAGGTGCAGGTTTTGATTTCAGACTGGTAAATATTTCAAA
TATCTATCATCATTTGCAATTAAAGGACTTTAGAAATGAAGAAGTTTTAAATTTTGACTTGATTGAATACATGCTTAAAA
ATTTGGAGTTGCAGAGTCGAGAAGAAAACTTGAGTGCTATCTTATTACAAGAAGATAACTTGGATTTTTTGGTTGAACTG
GCAAAGAAATTGTATGATAGTAAGTCTGAAAAATATAACTTGGAACAGTTTGGGCAGTTGATGGAGTTTTGGCTATCTAC
TAATCCACAAAGATTTATTGACTATATGAAGTCTACGAAGGTGGAGTATCAAGCTCCACTAAAGAATAAGTTCATTCGAG
CTTTGATGAATCAGGTGAACTTAGATAGTGTTGATAATGAAATAAAGGCGTTAGTAGCTCGTTATATCAATGAAAATAAT
GATTTAATTTCACCAAATGAAGATTTTACATCATTTTTCAAAACAAACCTTAAGGCAATCAATCTTAAATTCACTCATTTT
